# Complete Genome Sequence of Microcystis aeruginosa FD4, Isolated from a Subtropical River in Southwest Florida

**DOI:** 10.1128/MRA.00813-20

**Published:** 2020-09-17

**Authors:** Hidetoshi Urakawa, Taylor L. Hancock, Jacob H. Steele, Elizabeth K. Dahedl, Haruka E. Urakawa, Luka K. Ndungu, Lauren E. Krausfeldt, Barry H. Rosen, Jose V. Lopez

**Affiliations:** a Department of Ecology and Environmental Studies, The Water School, Florida Gulf Coast University, Fort Myers, Florida, USA; b Department of Marine and Earth Sciences, The Water School, Florida Gulf Coast University, Fort Myers, Florida, USA; c Department of Biological Sciences, Nova Southeastern University, Dania Beach, Florida, USA; Georgia Institute of Technology

## Abstract

We report the first complete genome of Microcystis aeruginosa from North America. A harmful bloom that occurred in the Caloosahatchee River in 2018 led to a state of emergency declaration in Florida. Although strain FD4 was isolated from this toxic bloom, the genome did not have a microcystin biosynthetic gene cluster.

## ANNOUNCEMENT

Lake Okeechobee is the largest lake in the southeastern United States (1,900 km^2^) and serves as a hub for water flow from the north to the Everglades in southern Florida. When flood control releases are necessary, water is directed to the Atlantic and Gulf coasts through two waterways, the St. Lucie River and the Caloosahatchee River, respectively (1). Since the 1980s, Lake Okeechobee and its waterways have suffered from chronic eutrophication problems and harmful cyanobacterial blooms (2). A recent Microcystis aeruginosa bloom that occurred in the Caloosahatchee River in 2018 led to a state of emergency declaration in Florida (3).

*M. aeruginosa* FD4 was isolated from surface water collected from the Caloosahatchee River at the Fort Denaud Bridge (26.7444N, 81.5103W) on 27 June 2018 during the bloom (3). The water quality parameters determined were as follows: water temperature, 32.4°C; pH 7.7; dissolved oxygen, 3.0 mg/liter; total iron, 1.32 mg/liter; chlorophyll *a*, 72.5 μg/liter; microcystin, 450.5 μg/liter; and hydrogen peroxide, 9.2 μg/liter (3). Surface scum was originally incubated with ultrapure water with pyruvic acid (8.8 μg/liter) at room temperature and then transferred to pyruvic acid-amended 10% BG-11 with germanium dioxide (10 mg/liter) and cycloheximide (100 mg/liter) to inhibit the growth of diatoms and other eukaryotes, respectively. Once we microscopically confirmed that the culture was unialgal, strain FD4 was maintained in BG-11 medium at 25°C under fluorescent light with a 12:12-h light/dark cycle. Genomic DNA was extracted using the Quick-DNA miniprep plus kit (Zymo Research). The DNA was made into SMRTbell libraries using the Express Template prep kit 2.0 (Pacific Biosciences). The sample was multiplexed with other samples into a single library and size selected using BluePippin (Sage Sciences) according to the manufacturer’s recommendations using the 0.75% DF Marker S1 high-pass 6-kb to 10-kb v3 run protocol and S1 marker (BPstart value of 8,000). The size-selected SMRTbell library was annealed and bound according to the SMRT Link setup and sequenced with the PacBio Sequel II system (Pacific Biosciences). Raw PacBio reads were converted to FASTA format with samtools fasta and then assembled with Flye version 2.6 (4). Default parameters were used for all software. The assembled genome was annotated with Prokka version 1.11 (5). The genome was annotated with SEED Viewer (6) and the NCBI Prokaryotic Genome Annotation Pipeline (GeneMark S-2+ version 4.10) (7). The final assembly of the genome comprised 5.45 Mbp at 125-fold coverage (*N*_50_ and *N*_90_, 10,352 and 6,453 bp, respectively) and consisted of two completely closed contigs, one chromosome, and one plasmid (Table 1).

**TABLE 1:** Genome statistics of Microcystis aeruginosa FD4^*a*^

Attribute^*b*^	Value
Total genome size (bp)	5,493,112
Chromosome size (bp)	5,449,501
Plasmid size (bp)	43,611
G+C content (%)	42.59
Total no. of genes	5,455
Total no. of CDSs	5,403
No. of coding genes	4,679
No. of CDSs with protein	4,679
No. of RNA genes	52
No. of rRNA sets (5S, 16S, 23S)	2
No. of tRNAs	42
No. of ncRNAs	4
Total no. of pseudogenes	724
No. of CRISPR arrays	3

a Annotation is based on the total genome and the NCBI Prokaryotic Genome Annotation Pipeline.

b CDS, coding sequences; ncRNAs, noncoding RNAs; CRISPR, clustered regularly interspaced short palindromic repeat.

Although strain FD4 was isolated from the toxic algal bloom (3), the genome did not have a microcystin biosynthetic gene cluster. It was confirmed by the annotations of the predicted open reading frames and homology searches against the genome. The presence of nine secondary metabolite gene clusters, including piricyclamide (8), micropeptin, and aeruginosin, were identified using antiSMASH version 5.1.2 (9). These numbers were quite small in comparison with those of *M. aeruginosa* NIES-2481 (GenBank accession number CP012375.1), in which 28 secondary metabolite gene clusters were found (10). Haft et al. (11) found that all bacteria with a short C-terminal homology domain that includes a highly conserved motif proline-glutamate-proline triad (PEP-CTERM) have both an outer membrane and exopolysaccharide production genes. Notably, 62 clusters of PEP-CTERM sorting domain-containing protein were found along with genes of exopolysaccharide biosynthesis polyprenyl glycosylphosphotransferase, polysaccharide pyruvyl transferase (CsaB), and WecB/TagA/CpsF family glycosyltransferase in the genome, suggesting the possible association of these genes for *Microcystis* colony formation (11). Consistent with the colony-forming ability of strain FD4, we found a coding gene of gas vesicle protein GvpC and *psb* and *apc* photoregulation clusters, which confer an ecological advantage to M. aeruginosa FD4 to compete with other phytoplankton through surface scum formation (12). Kardinaal and colleagues (13) reported that nontoxic strains of *Microcystis* were better competitors for light than toxic strains. Further genome annotation and genome comparisons with other strains of *M. aeruginosa* will provide additional insights into the ecological adaptation of this cyanobacterium.

### Data availability.

The genome sequence information has been deposited under BioProject number PRJNA595771 (GenBank accession numbers CP046973.1 [chromosome] and CP046974.1 [plasmid]). The PacBio reads have been deposited in the SRA under the accession number SRR12188899.
